# Quantum-enhanced wafer failure prediction in semiconductor fabrication

**DOI:** 10.1371/journal.pone.0361400

**Published:** 2026-11-10

**Authors:** Chenchu Sai Sashank Kapuluru, Srikumar Kukunuri, Sunil Chinnadurai, Rajak Shaik, Venkata Ramesh Naganaboina, G. Pradeep Reddy, You Sung Choi

**Affiliations:** 1 Department of Electronics and Communication Engineering, School of Engineering, Amrita Vishwa Vidyapeetham, Amaravati, Andhra Pradesh, India; 2 Department of Electronics and Communication Engineering, School of Engineering and Sciences, SRM University-AP, Amaravati, Andhra Pradesh, India; 3 Manipal Institute of Technology, Manipal Academy of Higher Education, Manipal, Karnataka, India; 4 Department of Software of Humanitas College, Kyung Hee University, Dongdaemun-gu, Seoul, Republic of Korea; Dr Shakuntala Misra National Rehabilitation University, INDIA

## Abstract

In recent years, rapid advancements in semiconductor manufacturing have underscored the critical need for robust quality control to maintain high yields and reliability in wafer production. In this context, accurately predicting wafer failures plays a pivotal role across the industry in minimizing production losses and enhancing cost efficiency. However, despite the use of traditional machine learning and deep learning methods to predict wafer failures, they have failed to achieve the desired levels of accuracy. Consequently, the present study investigates the application of Quantum Machine Learning (QML) methods on the SECOM dataset. This dataset was balanced using the Synthetic Minority Oversampling Technique (SMOTE), wherein the QML models were implemented using the PennyLane library. In this study, comprehensive data pre-processing was performed on the SECOM dataset; subsequently, various QML algorithms such as Quantum Support Vector Machines (QSVM) and Quantum Random Forests (QRF) were implemented to classify wafers as ‘good’ or ‘bad.’ Although initial improvements in performance compared to traditional methods were modest, leveraging PennyLane’s capabilities and fundamental principles of QML, this study highlights their transformative potential for industrial applications.

## 1. Introduction

Due to the significant financial losses caused by undetected wafer defects, the manufacturing of semiconductor wafers has become highly challenging amidst rising demand and the imperative for high yields [1]. To facilitate wafer yield inspection and support the study of semiconductor manufacturing processes, the SECOM dataset was introduced to researchers [2]. Researchers have applied machine-learning techniques to semiconductor manufacturing datasets for process monitoring and defect prediction, aiming to enhance reliability and reduce production losses [3–5]. However, researchers were unable to achieve high levels of accuracy using traditional data analysis methods. Consequently, the current research employs Quantum Machine Learning (QML) techniques to enhance the accuracy of wafer yield prediction.

In this direction, an initial examination of the SECOM dataset revealed the presence of ‘class imbalance.’ Researchers worldwide are working to address this issue of imbalanced data; research reviews have also been published, outlining methods for handling imbalanced data and the challenges that need to be addressed to further advance the field of ‘imbalanced learning’ [6,7]. To tackle this issue in SECOM dataset, the ‘Synthetic Minority Over-sampling Technique’ (SMOTE) was employed; this is one of several beneficial methods used to improve class distribution by achieving balance between the classes [8–10]. Subsequently, on one hand, various machine learning (ML) models – such as decision tree-based models [11], Support Vector Machines (SVMs, which utilize mathematical models capable of capturing subtle data patterns) [12], Random Forest (RF) algorithms, K-Nearest Neighbors algorithm [13] and neural networks [14,15] – have been applied to assess wafer failure; however, these algorithms demonstrated reliable performance when trained on clean datasets, they continued to face challenges when dealing with high-dimensional and noisy semiconductor data. On the other hand, ensemble models were combined with feature selection techniques to improve predictive performance while simultaneously reducing the computational load [16,17]. Following these research efforts, the application of clustering algorithms to detect anomalies in semiconductor systems was limited in terms of widespread deployment in large-scale industrial settings due to scalability constraints [17,18]. Although some models offered good accuracy, they faced scalability issues due to the presence of 590 features.

Hybrid quantum–classical systems have emerged as promising machine-learning frameworks for near-term quantum hardware [19–21]. In particular, parameterized quantum circuits can be viewed as expressive machine-learning models. A research review focusing on QML algorithms was published, highlighting QML’s ability to process complex, large-scale datasets with improved speed and efficiency [22]. The PennyLane library was developed, which is an open-source platform designed to integrate quantum and classical computing for practical QML experiments [23]. Using the PennyLane library, researchers focused on simulating quantum circuits to assess material defects, thereby strengthening the link between QML models and real-world manufacturing requirements [24]. Additionally, researchers explored the role of quantum entanglement in anomaly detection; however, progress was hindered by the technical limitations of Noisy Intermediate-Scale Quantum (NISQ) devices [25]. Despite advancements in QML, challenges remain regarding data preparation and the handling of real-world datasets within the constraints of limited qubit resources; consequently, hybrid approaches combining quantum models with classical preprocessing have been implemented [26]. In particular, recent work has explored hybrid quantum architectures for wafer defect detection [27] and quantum-enhanced data-driven learning for defect reduction in semiconductor etching processes [28], providing further context for the application of QML to semiconductor manufacturing.

This research advances the state of the art by evaluating QML-based methods for wafer failure prediction using the SECOM dataset. Initially, data imbalance and high dimensionality were addressed through the application of SMOTE and Principal Component Analysis (PCA); subsequently, quantum circuits ranging from 4 to 12 qubits were designed to enhance classification performance. To assess the practical efficacy of QML techniques in predicting industrial semiconductor defects, Quantum Support Vector Machines (QSVM) and Quantum Random Forests (QRF) were compared against traditional machine learning models.

## 2. Materials

### 2.1. PennyLane library

Quantum computing is a vast field where physical hardware is often scarce or inaccessible. To overcome this limitation, the proposed approach utilizes PennyLane – an open-source Python library developed by Xanadu, which is specifically designed to facilitate hybrid quantum-classical computing [23]. Serving as a crucial bridge between quantum simulators and classical machine learning frameworks, PennyLane acts as an exceptionally effective tool for both QML research and industrial prototyping. The PennyLane library employs “quantum nodes,” which are Python functions that encapsulate quantum circuits. These circuits are constructed using quantum operations such as rotation gates (e.g., RX, RY, RZ) and entangling gates (e.g., CNOT). Within these circuits, data can be mapped onto qubit states using techniques like angle embedding; this process prepares the qubits for quantum processing by rotating them based on input features. Furthermore, the PennyLane library integrates seamlessly with traditional machine learning frameworks such as PyTorch and TensorFlow. This allows quantum circuits to function as layers within classical neural networks, where quantum outputs (e.g., expectation values derived from qubit measurements) serve as inputs for subsequent classical processing. PennyLane employs automatic differentiation to compute circuit gradients, thereby enabling optimization via classical algorithms such as gradient descent. The library also supports various backends, including state-vector simulators – such as ‘default.qubit’ for ideal environments and ‘default.mixed’ for simulating the inherent noise found in NISQ hardware. It interfaces directly with physical quantum processors from vendors like IBM and Rigetti, although simulators remain prevalent in research due to current hardware limitations. Finally, PennyLane is particularly effective for variational quantum algorithms, in which circuit parameters are iteratively adjusted to minimize a cost function. This paradigm serves as the foundation for QML, closely mirroring the training processes of classical neural networks. In our current research study, PennyLane’s PyTorch interface was utilized to construct and optimize quantum circuits ranging from 4 to 12 qubits. A standard circuit implementation involves encoding pre-processed SECOM features via qubit rotations, applying entangling layers to capture complex feature correlations, and measuring the results to generate predictions. This hybrid approach leverages PennyLane to explore the benefits of quantum computing while relying on classical systems for training stability and scalability.

### 2.2. Quantum machine learning

Quantum Machine Learning combines the principles of quantum computing with classical machine learning techniques to address problems that are computationally intractable for classical systems; its application in wafer failure prediction stems from its theoretical capability to process the high-volume, complex data inherent in semiconductor manufacturing. Unlike a classical bit, which is restricted to a binary 0 or 1, QML utilizes fundamental quantum mechanical properties to process information. QML employs a qubit, which leverages the principle of superposition, i.e., a simultaneous linear combination of two states (for example, |ψ⟩=α|0⟩+β|1⟩, subject to the condition $|\alpha {|}^{\text{2}}+|\beta {|}^{\text{2}}=\text{1}$). This characteristic enables quantum systems to simultaneously represent multiple states. For instance, an 8-qubit system can simultaneously represent ${\text{2}}^{\text{8}}=\text{256}$ states, thereby providing a highly efficient mechanism for encoding large datasets. Furthermore, entanglement establishes profound connections between qubits, such that – regardless of physical proximity – the state of one becomes inextricably linked to that of another. In QML, entanglement gates (such as CNOT) harness these correlations to preserve and extract subtle, multidimensional signals within wafer characteristics – signals that traditional methods might otherwise overlook. Finally, quantum states are subject to constructive or destructive interference – a process that amplifies optimal computational pathways while simultaneously canceling out noise. QML algorithms leverage this phenomenon to enhance signal detection – a capability that is particularly useful for identifying rare events, such as wafer defects. QML adapts traditional machine learning workflows into a quantum environment through various approaches. Quantum-classical hybrids embed quantum circuits within classical deep learning layers, where data is encoded into qubits, processed by parameterized quantum gates, and measured. The resulting expectation values are then fed to classical optimizers, making this approach highly practical for near-term NISQ hardware [19]. Alternatively, while traditional Support Vector Machines rely on kernel functions to measure data similarity, QML utilizes quantum circuits to compute these kernels (e.g., $K(x_{i},x_{j})=|\langle x_{i}|x_{j}\rangle {|}^{\text{2}}$). This enables the model to map data into exponentially larger Hilbert spaces, thereby offering the potential to capture complex, non-linear patterns that are inaccessible to traditional kernels. Beyond classification, quantum circuits can be trained as generative models to learn from and sample complex probability distributions. In industrial contexts, this capability can be leveraged to simulate synthetic wafer defect patterns for the purpose of anomaly detection. Moreover, quantum clustering distance algorithms can efficiently estimate the similarity between an infinite number of data points, which facilitates unsupervised tasks such as grouping wafers based on unlabeled failure types [29].

In semiconductor manufacturing, predictive models must navigate the 590 complex features of the SECOM dataset to detect early signs of failure. While SVMs perform well with highly structured data, QML demonstrates significant capability in handling noise, high dimensionality, and non-linear relationships. Using PennyLane, this study empirically investigates this potential by benchmarking quantum circuits against classical baselines.

## 3. Quantum machine learning models

This section describes the QML models utilized in this research, outlining their operational principles, quantum enhancements, and their direct application to semiconductor wafer failure prediction.

### 3.1. Quantum support vector machine

The QSVM algorithm extends the classical SVM framework by transferring the kernel computation to a quantum circuit. In a classical SVM, functions such as the Radial Basis Function (RBF) map input data into higher-dimensional spaces to identify the optimal separating hyperplane. A QSVM replaces this mathematical projection with a quantum kernel. Input features are encoded into the physical states of qubits, processed by a quantum circuit, and measured to calculate the quantum fidelity between data points: $K(x_{i},x_{j})=|\langle \psi (x_{i})|\psi (x_{j})\rangle {|}^{\text{2}}$. The resulting kernel matrix is then optimized using classical SVM solvers.

Three distinct QSVM configurations were implemented using the PennyLane library. The first configuration, a 4 qubit QSVM, encodes 4 PCA-reduced features into 4 qubits utilizing Angle Embedding. It applies to a single layer of Basic Entangler Layers (CNOT gates) and computes the quantum kernel across a 500-sample subset. The second configuration expands the architecture to an 8-qubit QSVM using Angle Embedding accompanied by two entangling layers. These variant processes 500 samples using the lightning qubit state-vector simulator to accelerate computation. The final configuration, a 12 qubit QSVM, employs IQP Embedding (Instant Quantum Polynomial) to encode 12 principal components into 12 qubits across 1,000 samples. This model relies entirely on the complex implicit structure of the IQP embedding without requiring additional parameterized entangling layers.

The primary advantage of QSVM lies in its quantum kernel. It utilizes superposition to explore extremely high-dimensional feature spaces and entanglement to map complex, non-linear feature interactions. Theoretically, this capability enables QSVM to construct decision boundaries that traditional kernels cannot efficiently replicate.

### 3.2. Quantum random forest

The QRF algorithm adapts the Classical Random Forest (CRF) ensemble method by integrating quantum computing steps to enhance classification accuracy. While CRFs rely on aggregating multiple decision trees trained on data subsets to prevent overfitting, the limitations of current NISQ hardware necessitate a more streamlined approach. Consequently, in this study, the QRF replaces the traditional ensemble with a single, highly expressive decision tree powered by a quantum kernel. In this architecture, a quantum circuit projects classical data into a quantum Hilbert space using Angle Embedding, transforming four PCA-reduced SECOM features into a 4-qubit state. This circuit measures the fidelity between these states to generate a quantum similarity matrix. This matrix serves as the primary input for a classical decision tree, which employs standard splitting criteria (e.g., Gini impurity) to classify wafers. The QRF model applied to the SECOM dataset aims to distinguish between defective and non-defective wafers by leveraging the expressive power of the quantum feature space. The quantum-derived similarity metric possesses the capability to reveal subtle sensor correlations that traditional feature selection methods might obscure. Although the current scalability of this model is limited by its reliance on a limited number of qubits, the successful integration of a quantum kernel within a traditional decision structure represents a pivotal step toward robust hybrid anomaly detection in manufacturing environments.

## 4. Methodology

This section describes the systematic approach employed in the current research study to develop and evaluate quantum-enhanced models for wafer failure prediction using the SECOM dataset. This methodology comprises three fundamental stages such as data preprocessing, model development, and model evaluation, which are illustrated in Fig 1. These stages address the inherent challenges of the SECOM dataset – including its high dimensionality (590 features) and class imbalance (approximately 93% pass and 7% fail samples) – as well as the computational constraints of quantum hardware, such as the limited qubit availability on NISQ devices. By integrating traditional preprocessing techniques with QML algorithms – implemented via the PennyLane library – this pipeline aims to enhance prediction accuracy while mitigating overfitting and noise-induced errors. Building upon the dataset characteristics and fundamental QML principles discussed in the preceding sections, the following subsections provide a detailed exposition of each stage.

**Fig 1:**
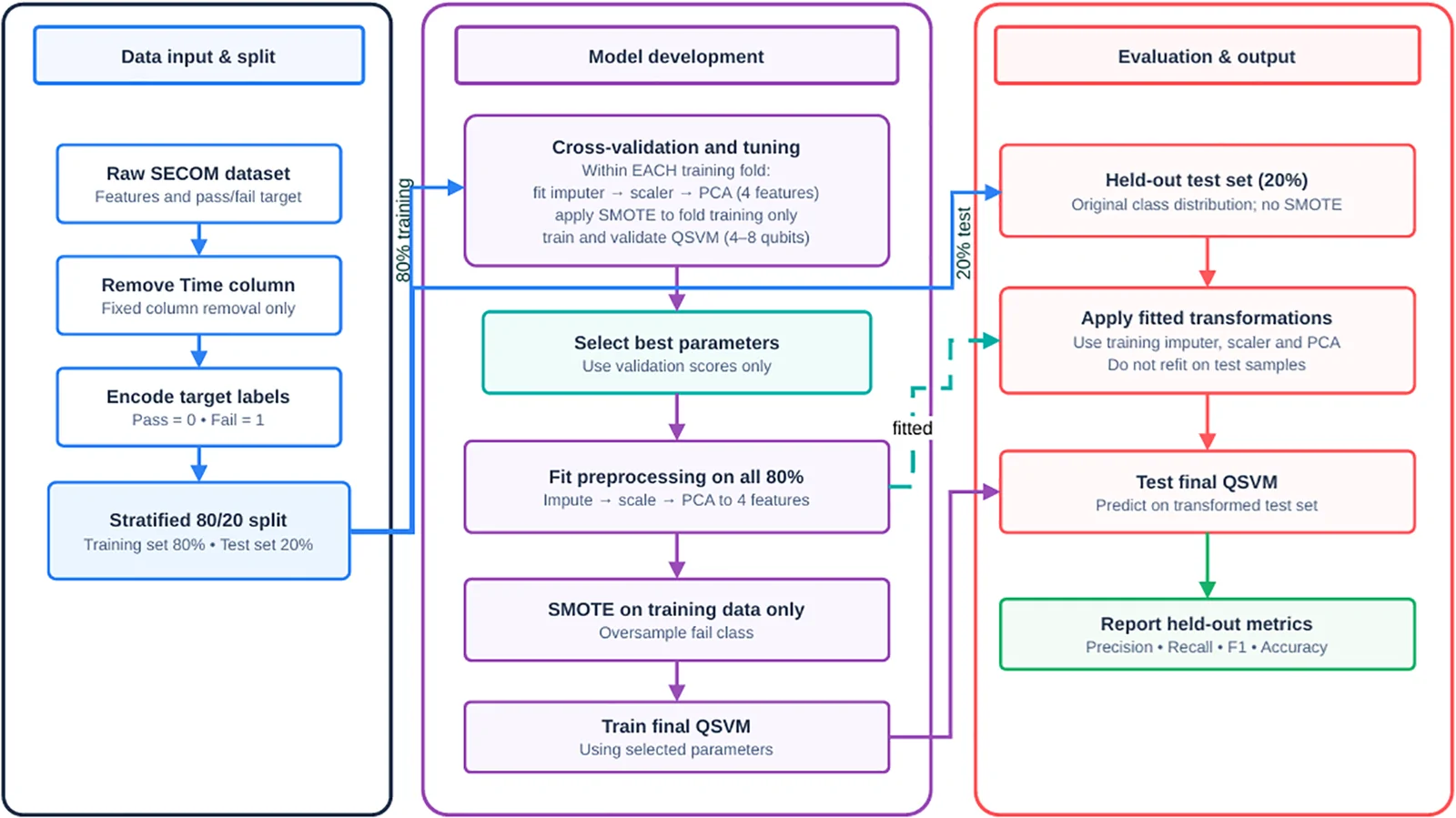
Schematic representation of the current research methodology.

### 4.1. Data preprocessing

In the initial phase, the raw SECOM dataset – comprising 1,567 benchmark samples collected from semiconductor manufacturing processes (which includes 590 numerical features and a binary’pass/fail’ label, where’-1’ denotes a pass and’1’ denotes a fail) – is transformed into a processed format suitable for QML models. This step is crucial for handling missing values, irrelevant features, class imbalance, and dimensionality. It ensures compatibility with qubit-constrained quantum circuits (e.g., 4–8 qubits). The preprocessing follows systematic workflow: imputation and cleaning, label conversion, class balancing, and feature reduction. Ultimately, this yields a balanced, normalized dataset for model training.

The raw SECOM dataset contains non-numeric elements and missing values (NaNs), which can introduce bias or computational errors during quantum encoding. Specifically, the’Time’ column – which records temporal metadata unrelated to failure patterns – was removed using Pandas’ df.drop()‘function to ensure the dataset consists entirely of numerical features. Missing values were imputed using ‘df.fillna(df.mean())‘, filling them with the column-wise numerical mean; this approach preserves the central tendency of each feature while preventing the introduction of artificial outliers. This imputation strategy aligns with standard practices for industrial datasets, where domain knowledge suggests that mean values effectively estimate unobserved sensor readings within semiconductor processes. Following imputation, low-variance features – defined as those exhibiting minimal fluctuation and thus possessing limited discriminative power – were filtered out using Scikit-learn’s Variance Threshold (set at 0.01), thereby reducing the feature count from 590 to approximately 312. This thresholding process enhances computational efficiency by eliminating redundant attributes – such as static sensor outputs – without incurring significant loss of information.

The original’Pass/Fail’ labels (’-1’ for Pass,’1’ for Fail) have been converted to a binary scheme (’0’ for Pass,’1’ for Fail). This facilitates compatibility with QML classification methods, particularly those relying on probabilistic outputs derived from quantum measurements. This remapping simplifies the target variable for algorithms such as QSVM, where binary decisions align with qubit state projections (|0⟩ for Pass, |1⟩ for Fail). This conversion ensures semantic consistency with classical baselines while simultaneously preparing the data for angle embedding within PennyLane Q Nodes, where labels can be encoded as rotation angles for variational optimization.

The SECOM dataset’s severe imbalance (93% pass samples versus 7% fail) predisposes classical and quantum models to bias toward the majority class, yielding poor recall for rare failure events critical in semiconductor fabrication. To mitigate this, the SMOTE is applied using the imbalanced-learn library (imblearn.SMOTE). SMOTE generates synthetic minority class samples by interpolating between existing failing instances and their k-nearest neighbors (typically k = 5), effectively oversampling the failure class to achieve balance (e.g., 1,463 samples per class). Unlike random oversampling, this method preserves the underlying data manifold and has been shown to improve prediction accuracy in imbalanced industrial contexts by balancing class distributions without excessively increasing the dataset size. The resulting resampled dataset exhibits a contrast between the original (skewed) and resampled (balanced) distributions. This enables balanced learning across classes, which is particularly beneficial given QML’s sensitivity to data sparsity – a characteristic stemming from superposition and entanglement.

High-dimensional data (even after variance thresholding) exceeds the qubit limitations of NISQ devices; therefore, reducing the dimensionality to 4–12 principal components is necessary to ensure quantum circuit feasibility. To achieve this, PCA is employed via Scikit-learn. This transforms the 312 features into a lower-dimensional subspace that retains 95% of the variance while adhering to qubit constraints (for instance, one feature per qubit via angle embedding). Fundamentally, PCA decomposes the covariance matrix to identify orthogonal components that capture maximum variance, thereby mitigating the “curse of dimensionality” and reducing noise propagation within quantum kernels. To ensure the scale invariance required for quantum rotations, the features are standardized using a Standard Scaler, centering them at zero mean and unit variance. This standardized, reduced dataset (the processed SECOM data) serves as the input for the subsequent model development phase, balancing fidelity to the original semiconductor process signals with the practical feasibility of quantum hardware.

### 4.2. Model development

This stage prioritizes an 80/20 train-test split, hyperparameter tuning, and the deployment of qubit-efficient classifiers. To assess their potential advantages in identifying non-linear failure patterns, quantum approaches – specifically QSVM and Quantum Random Forests (QRF) – are compared against traditional ML techniques.

The processed dataset is split into 80% training and 20% testing subsets using scikit-learn’s ‘train_test_split’ function. Following the train-test split, the SMOTE technique was applied to the training dataset to achieve class balance, prevent data leakage, and attain more robust, generalized performance. The training set guides the parameterization of the quantum circuit, while the held-out testing set evaluates unbiased performance. This aligns with best practices for critical applications such as wafer yield prediction.

To optimize model performance, hyperparameter grids were explored using grid search or Bayesian optimization within PennyLane’s PyTorch interface. Specifically, to ensure a comprehensive evaluation of the parameter space, Grid Search (GridSearchCV) was ultimately adopted as the primary optimization strategy across all evaluated models. For the 8-qubit QSVM, the search space included the regularization parameter (C) ∈ {0.1, 1, 10, 100} and the number of entangling layers ∈ {1, 2, 3}; ultimately, C = 1.0 and 2 layers were selected. For the QRF, grid search evaluated maximum tree depth ∈ {3, 5, 7, None} and minimum samples split ∈ {2, 5, 10}, converging at a maximum depth of 5 and a minimum split of 2. The classical SVM baseline was similarly optimized regarding C and kernel types, ultimately settling on C = 1.0 with an RBF kernel. Key parameters include the number of qubits (4–12), circuit depth (e.g., 2–5 layers of RX/RY/RZ rotations and CNOT entanglers), learning rate (0.01–0.1 for gradient descent), and embedding strategies (e.g., angle embedding for feature-to-rotation mapping). The tuning process minimizes a cost function – typically binary cross-entropy loss – with respect to variational parameters, leveraging PennyLane’s automatic differentiation for efficient hybrid optimization. This step addresses NISQ-era challenges, such as limited circuit depth, by iteratively refining configurations to maximize the quantum advantage in exploring the feature space.

The QSVM and QRF algorithms were developed and compared; these algorithms were simulated on PennyLane’s default qubit device, configured for 4 qubits (extensible up to 12). QSVM employs a quantum kernel to map features into a higher-dimensional Hilbert space, thereby enhancing the separation of pass/fail classes through entanglement-induced correlations – a feat unattainable via traditional methods. QRF extends traditional Random Forests by replacing decision trees with quantum decision oracles, integrating predictions from multiple shallow circuits to ensure robustness against noise. In these models, circuits are constructed as Q Nodes: features are embedded via rotations, followed by entangling layers (e.g., CNOT chains) for classification, and the prediction values are derived from ⟨Z⟩ measurements. Adam or COBYLA solvers are utilized for optimization, requiring 100–500 iterations to converge on qubit states that represent the probabilities of failure. Trained on an 80% subset, these models leverage QML principles – such as superposition for parallel feature processing and interference for noise cancellation – thereby demonstrating superior performance compared to classical SVMs or RFs on SECOM’s complex, noisy patterns.

Additionally, an analysis of the computational overhead of quantum simulations reveals that while classical models (e.g., standard SVMs and decision trees) train in a fraction of a second, QML simulations on classical state-vector backends exhibit distinct scaling profiles: (i) Variational hybrid architectures O(N)): scale linearly with sample size (N), requiring 83.11 ± 0.42 seconds for 1,500 samples; and (ii) Kernel-based models ($O\left(N^{\text{2}}\right)$): scale quadratically due to pairwise fidelity evaluations. In traditional simulators, evaluating the kernel for 100 samples took 63.26 ± 0.57 seconds, whereas full-scale evaluations (~3,000 resampled instances) resulted in severe bottlenecks exceeding 10,500 sec, necessitating data subsetting (500 samples) to ensure tractability (~18 min). Furthermore, while traditional simulators suffer from exponential state-vector memory scaling $(O\left({\text{2}}^{Q}\right)$ for Q qubits), native Quantum Processing Units (QPUs) can theoretically compute these kernel inner products efficiently, highlighting the trade-off between current simulation bottlenecks and future hardware acceleration.

## 5. Results and discussion

This section presents a performance analysis of several QML models utilized to predict wafer failures based on SECOM data; in addition to summarizing the empirical results, it provides an analysis of their general implications. The models employed in this research work comprise various variants of QSVM and QRF, which were developed using the PennyLane library. Furthermore, with the aim of establishing a performance baseline, a conventional SVM model was also evaluated for comparative purposes.

To investigate the scaling effects of qubits on classification capability and the impact of quantum embedding schemes, Fig 2 illustrates the performance metrics of the QSVM when the quantum circuit size is increased from 4 qubits to 8 qubits. In this context, prior to applying the QSVM to the dataset, the SMOTE was applied to address class imbalance in the classification models; Principal Component Analysis was used for dimensionality reduction; and Grid Search CV was employed to optimize hyperparameters. Fig 2 demonstrates that increasing the qubit count from 4 to 8 leads to a significant change in the QSVM’s performance metrics. On one hand, a 4-qubit QSVM – utilizing Angle Embedding and 500 entangled samples (reduced to 4 dimensions via PCA, which captures 94.59% of the variance) – yielded a baseline quantum testing accuracy of 62.00%. The computation of the quantum kernel for this model took 18 min and 17 sec. On the other hand, utilizing 1,000 samples (reduced to 8 features via PCA), an 8-qubit QSVM equipped with IQP Embedding – configured to capture 98.40% of the variance as its primary selection – demonstrated enhanced predictive power. This configuration achieved a testing accuracy of 73.00% and an impressive precision score of 0.9623.

**Fig 2:**
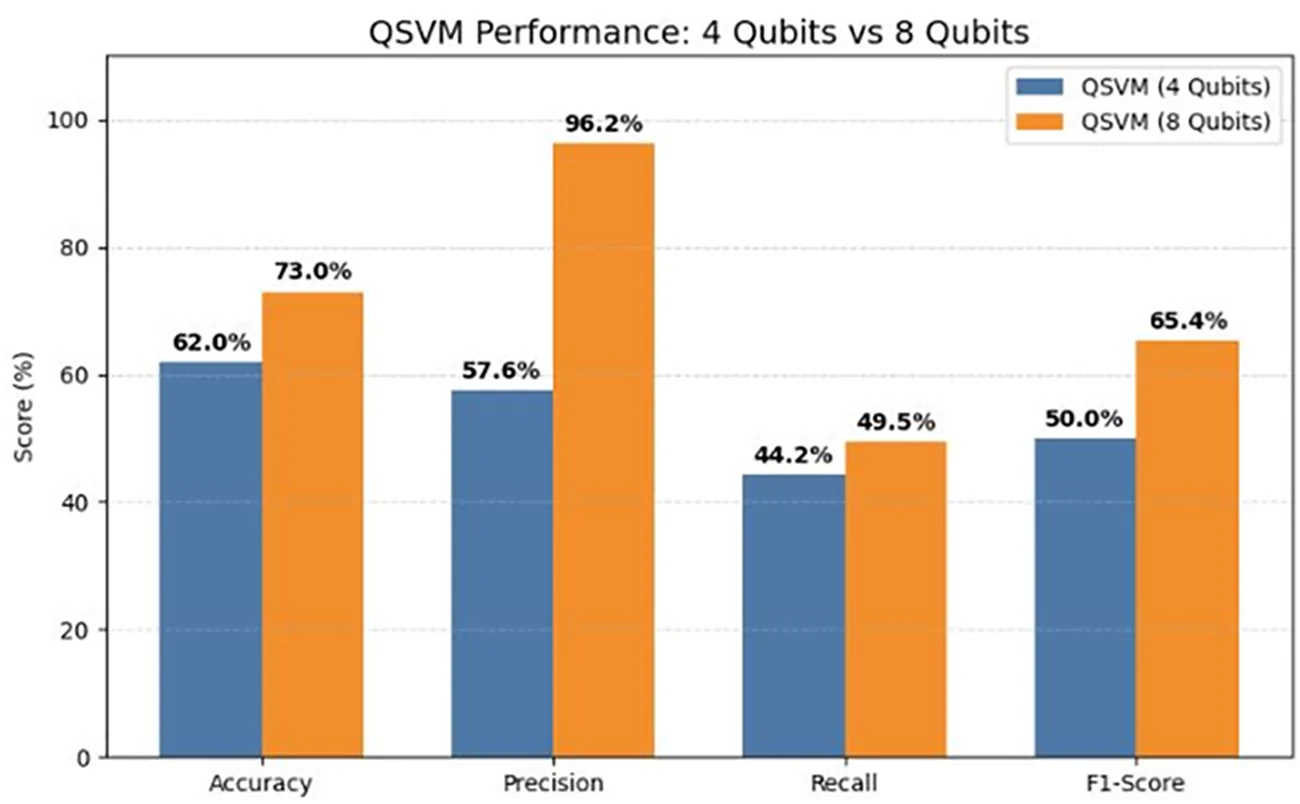
Comparison of QSVM performance metrics (4 Qubits vs. 8 Qubits).

Subsequently, the performance of the traditional SVM method was compared with that of the QSVM; the results for the QSVM models and the traditional SVM baseline are shown in Fig 3. While the traditional SVM achieved an accuracy of only 44.00% due to the high dimensionality and complex non-linearities in the data, the 8-qubit QSVM demonstrated significantly better accuracy than the traditional SVM. This implies that by leveraging the principles of quantum kernel superposition and entanglement, it was possible to project the dataset’s features into a high-dimensional Hilbert space, where they were more clearly separable. The empirical findings regarding these models were examined in conjunction with an analysis of their broader implications. The models considered in this study included variations of QSVM and QRF, both of which were developed using the PennyLane library. A traditional SVM was also tested to serve as a performance baseline. To further assess the statistical stability of the QSVM model the classifier was tested using both 4-qubit and 8-qubit feature maps. The 8-qubit model performed better overall, achieving an accuracy of 73.0%, precision of 96.2%, recall of 49.5%, and an F1-score of 65.4%, compared with 62.0% accuracy, 57.6% precision, 44.2% recall, and a 50.0% F1-score for the 4-qubit model. Increasing the number of qubits from 4 to 8 improved the accuracy by 11.0 percentage points, precision by 38.6 points, and F1-score by 15.4 points, while recall increased only slightly by 5.3 points. The very high precision of the 8-qubit model shows that it was effective at avoiding false-positive predictions. This trade-off between precision and recall also explains why the 8-qubit QSVM achieved an F1-score of 65.4%.

**Fig 3:**
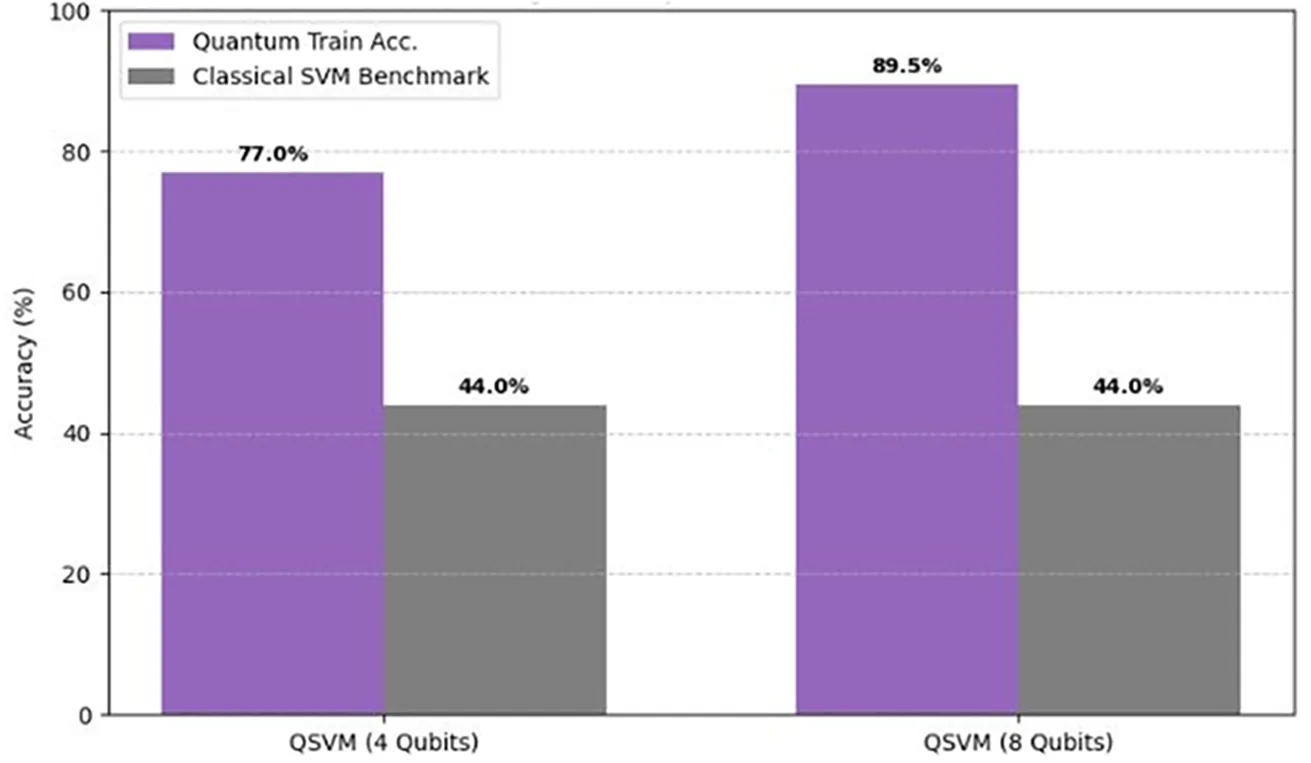
Performance comparison between the QSVM models and the classical SVM baseline.

In addition to the QSVM, a quantum kernel-based QRF model utilizing a traditional decision tree structure was implemented on the SECOM dataset, and its performance metrics are presented in Fig 4. For the QRF model, a 4-qubit angle-embedding method was employed to compute the quantum similarity matrix, which was subsequently fed into a classical classifier. The QRF model was trained using a set of four PCA-reduced features, yielding a test accuracy of approximately 60.00%, a precision of 0.620, and a recall of 0.520. To further assess the statistical stability of the QRF model, this configuration was evaluated across five additional independent runs; this evaluation yielded an average F1-score of 0.7240 ± 0.1133, an average ROC-AUC of 0.7385 ± 0.0976, an average precision of 0.6939 ± 0.0901, an average recall of 0.7622 ± 0.1519, and an average accuracy of 73.68% ± 9.56. The reported standard deviations provided clear empirical evidence of the model’s algorithmic stability and predictive reliability across repeated trials. Although the QRF model outperformed the classical SVM baseline, it lagged behind the QSVM architecture. These results suggest that, compared to QSVM’s maximum-margin approach, relying on a single decision tree parameter (even when parameterized via a quantum kernel) is less effective for establishing robust decision boundaries for this specific dataset. While the proposed framework demonstrates the potential of QML for wafer failure prediction, it is constrained by NISQ limitations. Currently, limited qubit scalability necessitates drastic dimensionality reduction (compressing 590 features into 12 principal components), which carries the risk of losing subtle defect indicators. However, this framework is designed to scale effectively during the transition to fault-tolerant quantum computing (FTQC). Future error-corrected architectures will bypass classical PCA, enabling the direct ingestion of high-dimensional raw sensor data. By employing state preparation techniques such as amplitude encoding, future versions will embed the entire uncompressed dataset directly into a complex Hilbert space. This evolution eliminates classical information loss, thereby allowing quantum models to simultaneously map highly complex, non-linear correlations across hundreds of variables and securing their long-term industrial viability.

**Fig 4:**
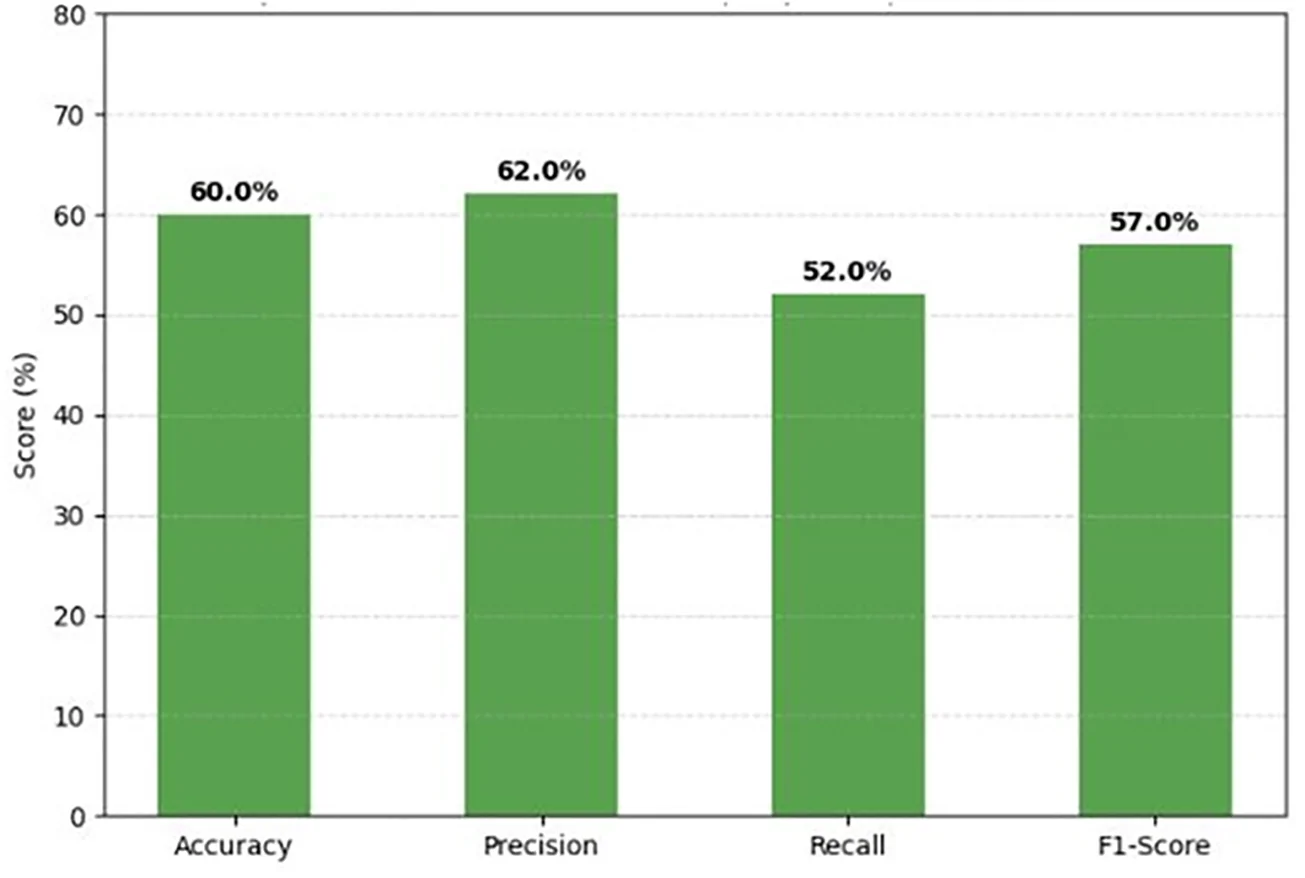
Performance metrics of the QRF model.

Finally, Fig 5 summarizes the comparison of performance metrics for two distinct models built upon the SECOM dataset: QSVM (4-qubit and 8-qubit) and QRF (4-qubit). Among these, the 8-qubit QSVM decisively delivers the most comprehensive classification performance. This aligns with the hypothesis that quantum kernels are capable of unearthing hidden correlations within large-scale industrial data. However, an examination of key metrics reveals significant limitations. Although the 8-qubit QSVM exhibits the highest precision, its recall stands at 0.495. This implies that the model still fails to identify a substantial portion of actual wafer failures (resulting in false negatives). In a semiconductor fabrication setting, undetected failures can lead to significant financial losses; consequently, recall serves as a critical performance metric. On the other hand, computation time remains a major impediment. The 8-qubit QSVM required over two hours for computation, highlighting the scaling challenges encountered when utilizing quantum circuit simulators (or Noisy Intermediate-Scale Quantum) devices. To accommodate qubit limitations, the SECOM dataset – comprising 590 features – had to be drastically reduced to just 4 or 8 principal components; this process may have resulted in the elimination of low-level indicator signals. Although quantum models have been demonstrated to outperform traditional models across certain metrics of accuracy and precision, these scalability and hardware constraints must be overcome to enable the deployment of quantum models in real-time wafer production systems.

**Fig 5:**
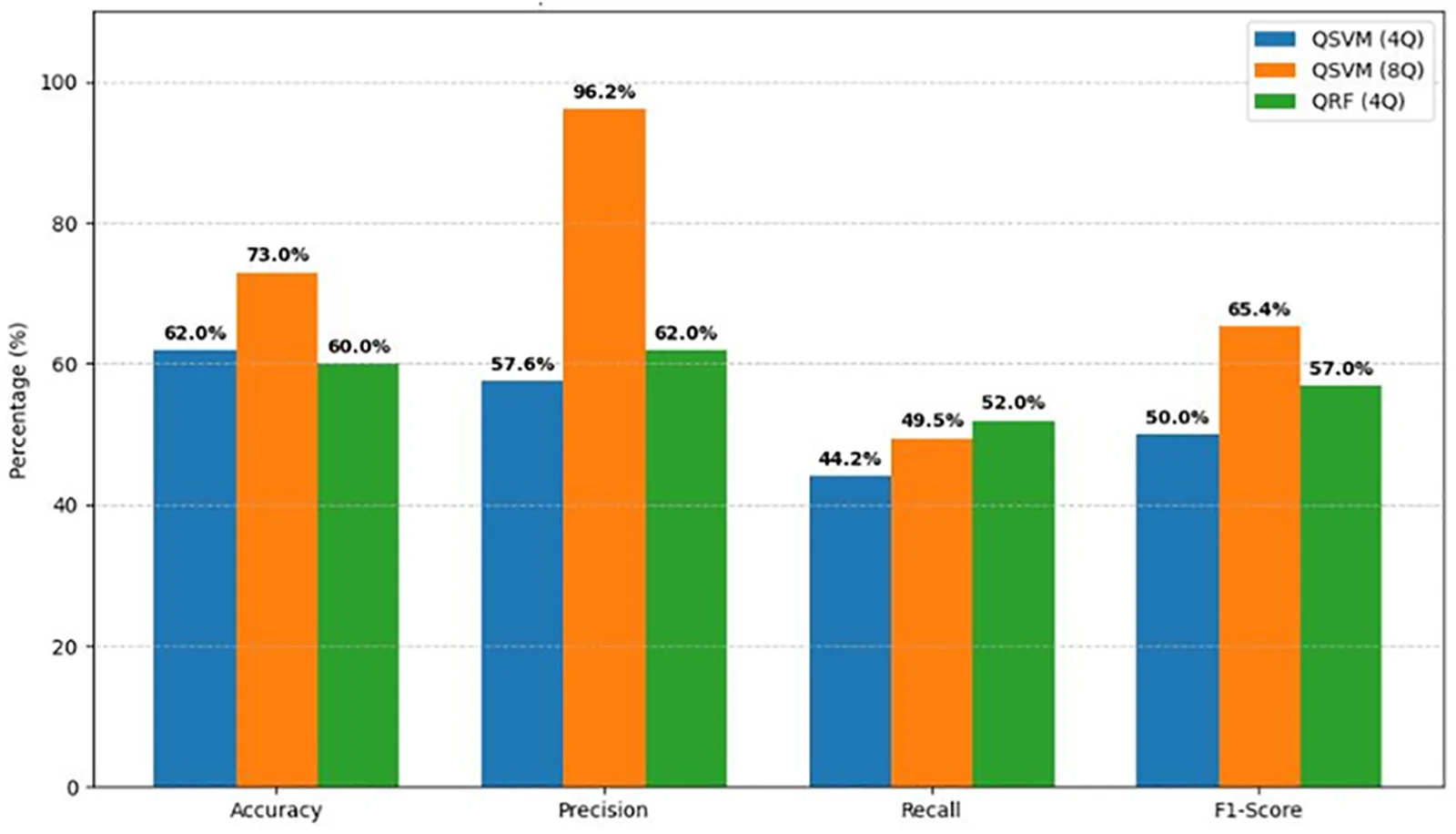
Overall comparison of performance metrics across the evaluated quantum and classical models.

## 6. Conclusion

In Summary, the current study leverages the PennyLane library to apply various QML techniques for predicting wafer failure. From early statistical methods to advancements in traditional ML, the field has progressed toward addressing high-volume, imbalanced datasets such as SECOM. In this work, our research advances this trajectory by implementing QSVM and QRF on the SECOM dataset; notably, an 8-qubit QSVM achieved an accuracy of up to 73%, surpassing traditional SVM baselines (44%). These results validate QML’s potential as a transformative tool for industrial applications, yet they also underscore persistent challenges. The limited number of qubits (4–8, versus 590 SECOM features) necessitated dimensionality reduction – thereby introducing a risk of information loss – while shallow circuit depths, relative to traditional deep learning, constrained model complexity.

Based on current research and with next-generation quantum hardware in mind, future efforts will focus on scaling the number of qubits beyond 50. This expansion will enable the preservation of a larger number of SECOM features, thereby offering the potential to surpass the accuracy standards of traditional machine learning. Furthermore, deep circuits incorporating error correction will enhance QML’s capability to model non-linear failure patterns. Integrating quantum error mitigation and advanced embeddings (beyond, for instance, IQP Embedding) can mitigate the effects of noise. Additionally, hybrid models that combine QML with traditional deep learning can bridge current gaps, while industrial partnerships can validate these approaches on real-time wafer production lines. By addressing these avenues, QML can evolve from a promising paradigm into a cornerstone of semiconductor manufacturing optimization.
